# Supplementary material for: Predictors and outcomes of recognition of intellectual disability for adults during hospital admissions: A retrospective data linkage study in NSW, Australia
Source: PLoS One. 2022 Mar 25;17(3):e0266051. doi: 10.1371/journal.pone.0266051 (PMC8956190; doi:10.1371/journal.pone.0266051)
Supplement: Attachment — Submitted filename: Response to Reviewers.docx [file pone.0266051.s003.docx]

| **Reviewer 2** | |
| --- | --- |
| 1) The intellectual disability basically develops at the young age which may affect the next ages. I am not sure that the participants (especially the elders) were asked whether they were developed a confirmed ID syndrome in their early stages of life (any medical confirmation about that??). Author should clear these although they mentioned autism spectrum disorder (ASD) which is a developmental disability that can cause significant social, communication and behavioral challenges. | Our project did not involve the collection of data directly from participants through interviews – all the data used in the project was acquired via administrative datasets (which included all relevant diagnostic flags, including those for intellectual disability and autism spectrum disorder). We have attempted to clarify this point in our Methods section on page 6, where we have added the following section in italics to paragraph 1:  “This study was a retrospective cohort study, conducted as a substudy within a bigger data linkage infrastructure project described in Reppermund et al.[22] *All data used in this study were collected into administrative datasets during the interaction of members of the cohort with administrative services (e.g., hospital or disability services). No participant data were gained through direct interaction with members of the cohort, such as via structured interviews.* Linkage was performed by the NSW Centre for Health Record Linkage using best practice methods.” |
| 2) Another point is that the elderly people are more likely to develop Dementia/Alzheimer disease. I would like to request to add this type of description whether the participants had their diagnosis of Dementia/Alzheimer disease before?? Although the participants were asked about mental disorder. | We have carefully considered this point, and based on the combined experience of the team in both the study of intellectual disability and dementia, believe that the addition of a discussion around dementia would not be relevant to the current paper.  Our reasoning is that dementia and intellectual disability represent different groups of disorders, and based on experience with dementia and intellectual disability in other projects, we believe dementia would not present a confounding factor to the recognition of intellectual disability in hospital settings. |
