# Supplementary material for: Predictors and outcomes of recognition of intellectual disability for adults during hospital admissions: A retrospective data linkage study in NSW, Australia
Source: PLoS One. 2022 Mar 25;17(3):e0266051. doi: 10.1371/journal.pone.0266051 (PMC8956190; doi:10.1371/journal.pone.0266051)
Supplement: Attachment — Submitted filename: Reviewer comment.docx [file pone.0266051.s002.docx]

The article entitled “Predictors and outcomes of recognition of intellectual disability for adults during hospital admissions: A retrospective data linkage study in NSW, Australia” is representing the factors and outcome associated with the intellectual disability for adults during hospital admissions. The overall manuscript (MS) sounds good, well represented, and described. I think it will contribute more for the aged people related research. I would like to take attention of the authors to address/explain the following concerns for updating the MS before publication:

(i) The intellectual disability basically develops at the young age which may affect the next ages. I am not sure that the participants (especially the elders) were asked whether they were developed a confirmed ID syndrome in their early stages of life (any medical confirmation about that??). Author should clear these although they mentioned autism spectrum disorder (ASD) which is a developmental disability that can cause significant social, communication and behavioral challenges.

(ii) Another point is that the elderly people are more likely to develop Dementia/Alzheimer disease. I would like to request to add this type of description whether the participants had their diagnosis of Dementia/Alzheimer disease before?? Although the participants were asked about mental disorder.

The manuscript should have these explanations for its better quality and application.
